# A Memorable Membership Moment

**Published:** 1986-06

**Authors:** J. L. Burton

**Affiliations:** Consultant Dermatologist, Bristol Royal Infirmary, Bristol


### A memorable Membership moment

**J. L. Burton B.Sc., M.D., F.R.C.P.**
Consultant Dermatologist, Bristol Royal Infirmary, Bristol

Most unpleasant memories fade eventually, but some are so horrendous that one's psyche is branded for life. The clinical and oral examinations for the MRCP and FRCS diplomas seem to provide a rich source of such searing memories. Most hospital consultants have a 'Membership' or a 'Fellowship' story—here's mine.

I knew I'd done fairly well on the papers, and the clinical examination also went well. I was helped along by the fact that my major case, with bizarre neurological symptoms, was undiagnosable even by the consultants at the National Hospital for Nervous Diseases, who'd been investigating him for several weeks. Fortunately I'd just read a review of the hereditary sensory neuropathies, and could explain more or less convincingly why he didn't fit any of them. My luck held too with the minor cases—there was only one really difficult case, and he whispered the answer to me when the examiner was called away to the telephone. The memorable bit occurred when we came to the viva.

As I was ushered into the room I was confronted by a pair of rather peppery old gentlemen, one of whom appeared pained and the other seemed bored. The first looked at me with some disgust. 'Name?' he barked. 'Burton, sir'. 'Tell me about the importance of trace metals in medicine' he said. 'Yes sir', I said, hoping for inspiration, 'which ones?' 'All of them'.

Now it must be admitted that the trace metals had not figured largely in my preparation for the examination, but I had heard of a few of them, and with a certain amount of huffing, puffing, prompting and prodding, I eventually stumbled through them, hoping that the gradient would ease somewhat on the next leg.

The second examiner was a famous Guy's physician. He walked over to a viewing box, and when he put up a chest x-ray showing an obvious mediastinal mass, my spirits rose. At that time I was working at the Brompton Hospital, where I'd had excellent weekly tuition on radiographic interpretation from the great George Simon, and boy, did I know about mediastinal masses!

Unfortunately what I knew, and what the examiner seemed to be wanting me to tell him, in no way tallied. He grew increasingly exasperated and my suggestions became increasingly wild, as I tried in vain to fathom what the old goat was after. At length his patience was obviously exhausted and he turned away from me and went back towards the desk. As he walked away he started his next question. 'Suppose you're in general practice, and a young man walks into the surgery with a story of feeling unwell and feverish for about two days, and he's come to the surgery now because for the last few hours he can't swallow properly. What's the diagnosis?'

As he reached the end of this question, he turned to face me and immediately there was a great wet red chasm where his right eye had been two minutes ago. He leered and blinked encouragingly at me, so that as the eyelids sucked open, the moist red membranes in the empty socket flashed like a lighthouse in hell. I wondered where on earth he'd put his glass eye, and whether he had a pocket full of them, like enormous gob-stoppers.

'Well'? he said.

'Yes, er...sorry', I stammered. 'I think tonsillitis would be the first thing I should think of'.

'Rubbish', he said. 'Bulbar polio. You'd have missed the diagnosis and the patient would die'.

Editor's Note—Many of our readers will recall such encounters in their examination days. the Editor would be glad to consider them for a 'Memorable Moments' series.

